# Interspecies differences in the expression of cannabinoid receptors at the tissue and cellular levels

**DOI:** 10.4103/NRR.NRR-D-25-00806

**Published:** 2025-10-30

**Authors:** Sydney Lawley, Audrey Green, Cole Johnson, Michael D. Burton

**Affiliations:** Neuroimmunology and Behavior Lab (NIB), Department of Neuroscience, School of Behavioral and Brain Science, Center for Advanced Pain Studies (CAPS), University of Texas at Dallas, Richardson, TX, USA

**Keywords:** CB1R, CB2R, endocannabinoid system, human, mouse, rat, rodent, species differences, translational research

## Abstract

Understanding the cellular and molecular distribution of cannabinoids can address a highly contentious perspective in the pain neuroscience field: whether cannabinoids are viable in pain relief. Due to insufficient evidence for cannabinoids in reducing pain in clinical trials and gaps in knowledge across the translational research process, the International Association for the Study of Pain (IASP) published a position statement in 2021 recommending against the general use of cannabinoids to treat pain. A possible mechanistic reason for the lack of translatability is interspecies differences in the expression of the cannabinoid type 1 and type 2 receptors at the tissue and cellular levels. Additionally, the anatomical site that is most important for analgesia has been elusive. This review aims to provide a deeper understanding of the anatomical distribution of cannabinoid receptors throughout the nervous system across species. We traverse historical and contemporary literature to illustrate the progression of methodology and perspectives. We discuss co-localized markers on cannabinoid receptor-expressing cells to elucidate possible anatomically dependent roles of the endocannabinoid system. We also discuss differences in cannabinoid receptor expression across species that may contribute to challenges in translatability between rodents and humans. Lastly, we cover how various types of pain can differentially alter the expression of cannabinoid receptors and how this may impact cannabinoid-based therapeutics.

## Introduction

**Figure 1:**
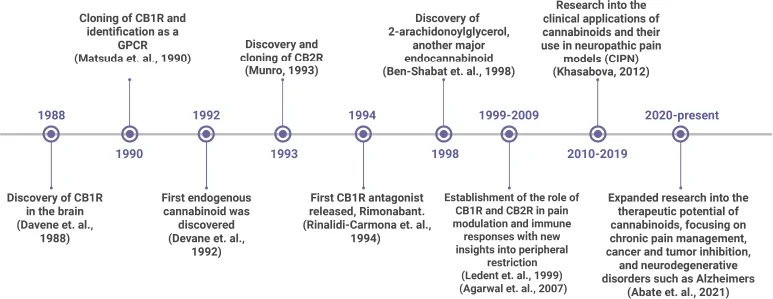
Timeline of key discoveries in cannabinoid research Timeline illustrating the significant discoveries in cannabinoid research. Created with BioRender.com. CB1R: Cannabinoid receptor 1; CB2R: cannabinoid receptor 2; CIPN: chemotherapy-induced peripheral neuropathy; GPCR: G protein-coupled receptors.

The massive disease burden associated with chronic pain is an international health crisis estimated to affect 20.9% of U.S. adults and 27.5% of adults worldwide (Zimmer et al., 2022; Rikard, 2023). Concerningly, more adults are developing chronic pain than are recovering, further increasing the burden (Nahin et al., 2023). Current chronic pain treatments fall short in several ways, including development of long-term tolerance, occurrence of adverse effects, and the potential for addiction and misuse. Thus, alternative compounds, such as exogenous cannabinoids, have been investigated as alternatives (**Figure 1**; Devane et al., 1988; Matsuda et al., 1990; Munro et al., 1993; Rinaldi-Carmona et al., 1994; Agarwal et al., 2007). Recently, focus has also been placed on the interactions between the endocannabinoid system and pain (Devane et al., 1992; Ben-Shabat et al., 1998; Ledent et al., 1999; Khasabova et al., 2012; Abate et al., 2021).

Cannabinoids are a class of compounds that bind to the G-protein coupled receptors, cannabinoid receptor 1 (CB1R) and cannabinoid receptor 2 (CB2R), to inhibit adenylyl cyclase, activate mitogen-activated protein kinase, and regulate calcium ion channels through the Gαo/Gαi signaling pathway (Howlett, 2002; Mackie, 2008; Lu and Mackie, 2021). CB1R is primarily expressed on interneurons, facilitating the fine-tuning of neuronal circuits and regulation of excitability, whereas CB2R is mostly restricted to non-neuronal cells, where it modulates immune responses (Galiègue et al., 1995; Howlett et al., 2002; Younts and Castillo, 2014). Since the discovery of CB1R and CB2R in 1988 and 1993, respectively, researchers have utilized multiple techniques to assess their anatomical distribution and cellular localization at the protein level (**Table 1**; Kraus, 1897; Caro and Van Tubergen, 1962; Moore, 1965; Hamilton and Suhr-Jessen, 1980; Von Behring and Kitasato, 1991; Bundy, 1997). Molecular techniques have also been used to understand factors influencing CB1R and CB2R gene expression (Gall and Pardue, 1969; Munro et al., 1993; Woodruff, 1998; Tang et al., 2021). Their ubiquitous expression in the nervous system and periphery facilitates the production of various biological and psychoactive effects, such as anti-nociception and analgesia.

**Table 1:** Summary of the several techniques used to quantitatively determine CB1R gene and protein expression in cells and tissue.

Technique	Description	Target	Pros	Cons
Autoradiography	Tissue sections incubated with [^3^H]CP55,940, exposed to film, and then developed and imaged.	Radioisotope-labelled CB1R	High sensitivity and spatial resolution. Decreased variability due to absence of antibodies and probes.	Non-specific binding, extensive time, and inability to determine expression at a cellular level.
Immunohistochemistry	Use of a primary antibody raised against an epitope of the protein and a secondary antibody with a conjugated fluorescent label.	CB1R Specific Antigen	High spatial resolution enabling colocalization studies with neurotransmitter markers to map CB1R distribution.	Concerns about CB1R antibodies leading to varied staining patterns and proportions of CB1R-immunoreactive cells in tissue sections.
Calcium binding proteins	Used as markers for interneurons and can identify specific neuron populations for CB1R co-localization.	Calcium ions within specific neuronal subtypes	Can monitor real time changes in CB1R activity, has high sensitivity, and can assess CB1R expression is specific cell-types.	Dependent on calcium concentrations in the environment and cell-type variability.
*In situ* hybridization	Radioactive, non-radioactive, or fluorescent labels are added to antisense probes complimentary to a coding region of CB1R cDNA or mRNA, which then hybridize with their respective base pair sequence.	*CB1R* mRNA	Direct detection of gene expression and provides insight into the localization of CB1R that may not be detected by immunohistochemical techniques after undergoing protein phosphorylation and degradation.	Low sensitivity to genes expressed at low levels and complexity of the procedure.

CB1R: Cannabinoid receptor 1; cDNA: complementary deoxyribonucleic acid; mRNA: messenger ribonucleic acid.

The pain-relieving properties of cannabinoids have been well established in preclinical models utilizing a wide variety of endocannabinoid system targets in different pain models (Starowicz and Finn, 2017; Milligan et al., 2020; Slivicki et al., 2022). However, clinical studies have been unable in consistently reproduce these effects; for example, the peripheral cannabinoid agonist AZD1940 did not reduce acute pain or hyperalgesia after dental surgery or capsaicin administration (Kalliomäki et al., 2013a, b). Systematic reviews of clinical studies have ranged from finding negative to moderate evidence for short-term cannabinoid treatments in relieving chronic pain (Andreae et al., 2015; Whiting et al., 2015; Häuser et al., 2018; Haroutounian et al., 2021). However, high risk of bias, poor reporting, and low-quality evidence of clinical trials has rendered the current body of studies unable to ascertain the effectiveness of cannabinoids (Fisher et al., 2021; Moore et al., 2021). Thus, the IASP concluded that, despite there is compelling evidence for cannabinoid-based analgesia in preclinical models, there is not an empirical basis for the use of cannabinoids for pain relief due to the gap in preclinical to clinical translatability (IASP Presidential Task Force on Cannabis and Cannabinoid Analgesia, 2021).

It is imperative to further develop our understanding of the endocannabinoid system across species, cell populations, and pathologies to address these obstacles. For example, studies have challenged the belief that CB1R and CB2R are solely localized to the nervous system and periphery, respectively (Galiègue et al., 1995; Ryberg et al., 2005; Atwood and Mackie, 2010; Veress et al., 2013; Grabon et al., 2023). Additionally, the anatomical location of cannabinoid receptors critical for analgesia must be discovered to avoid nonspecific effects such as impaired cognitive functioning, hypothermia, catalepsy, tachycardia, inhibited gut motility, and orexia (Ameri, 1999; Izzo and Sharkey, 2010; Vianna et al., 2012; Koch et al., 2015; Castorena et al., 2021).

**Figure 2:**
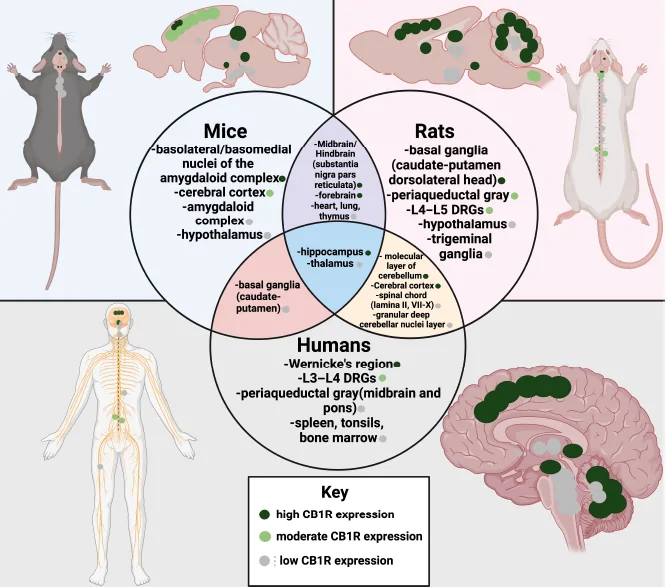
Comparison of CB1R expression levels in the central and peripheral nervous systems of mice (blue), rats (pink), and humans (green). High CB1R expression is indicated by a dark green circle, moderate expression by a lighter green circle, and low expression by a gray circle or gray dashed line, reflecting the varying distribution of CB1R across species and neural locations. The areas of the nervous system are ordered in descending order of CB1R expression. The purple region indicates similarities in expression between rats and mice, the yellow region between rats and humans, and the orange region between humans and mice. The darker blue region in the center highlights areas of similarity in CB1R expression among all three species. These are the known areas of similarity in CB1R expression identified through a literature search. Additional similarities may exist and warrant further investigation. Created with BioRender.com. CB1R: Cannabinoid receptor 1; L3: lumbar 3; L4: lumbar 4; L5: lumbar 5.

This review aims to provide a better understanding of the localization of cannabinoid receptors at the cellular and anatomical levels to guide more thorough and well-informed research on cannabinoids in the context of pain. Similarities and differences in cannabinoid receptor expression across rodents and humans are highlighted as aspects to consider in translational research (**Figure 2**; Herkenham et al., 1991; Glass et al., 1997; Tsou et al., 1998; Marsicano and Lutz, 1999). Additionally, expression changes induced by pain pathophysiology are examined to identify potential therapeutic targets.

## Search Strategy

The literature discussed in this review was retrieved using PubMed, Google Scholar, and Web of Science databases. Relevant studies on cannabinoid receptor expression in humans, rats, and mice were identified using a combination of the following keywords: “cannabinoid 1 receptor,” “cnr1,” “cannabinoid 2 receptor,” “cnr2,” “expression,” “localization,” and “distribution.” To ensure a range of historical and contemporary perspectives and methodologies were present, a date criterion was not used. The articles were published between 1897 and 2025. Additional literature was sought to provide insight into the implications of differences in cannabinoid receptor expression. Databases were used to find articles discussing related brain regions and cellular markers. The abstract and full text of all works were manually reviewed to ensure relevance to the review.

## CB1R Expression throughout Regions of the Central Nervous System across Different Species

### Cortex

**Figure 3:**
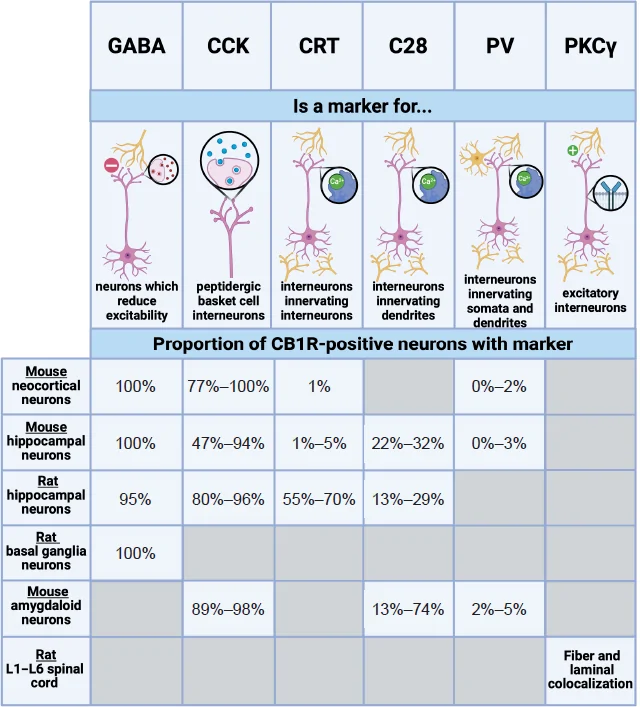
Graphical representation of the co-localization of CB1R with common neuronal markers in the central nervous system. A grey box indicates that the available literature does not report the proportion of CB1R-positive neurons expressing the specified marker. Created with BioRender.com. C28: Calbindin D-28K; CB1R: cannabinoid receptor 1; CCK: cholecystokinin; CRT: calretinin; GABA: gamma aminobutyric acid; PKCg: protein kinase C gamma; PV: paravalbumin.

Cortical *CB1R* gene and protein expression is similar between rodents and humans, with enrichment in the association, somatosensory, and motor cortices (Herkenham et al., 1991; Glass et al., 1997; Tsou et al., 1998; Pak et al., 2023). CB1R expressed in the associational corticies allows for modulation of high-level cognitive processes, including decision making and time perception (Tsao et al., 2018; Chauhan et al., 2021). Across species, the outermost cortical laminae (I & VI) are characterized by high levels of CB1R mRNA and protein (Herkenham et al., 1991; Glass et al., 1997; Tsou et al., 1998; Marsicano and Lutz, 1999). In contrast, the intermediate laminae have low *CB1R* gene and protein expression, with high heterogeneity between cortical regions (Herkenham et al., 1991; Glass et al., 1997; Tsou et al., 1998; Marsicano and Lutz, 1999). CB1R mRNA-positive mouse cortical neurons are localized to two GABAergic subpopulations: cholecystokinin-positive peptidergic basket cells and calbindin D-28K-expressing interneurons that form axodendritic synapses (**Figure 3**; Marsicano and Lutz, 1999). Widespread distribution of CB1R-expressing interneurons throughout the cortical layers facilitates multi-level modulation of afferent information from the thalamus and brainstem before it is relayed to other regions (Palomero-Gallagher and Zilles, 2015). CB1R action in the cortex contributes to various psychoactive effects that necessitate peripherally restricted CB1R agonists for analgesia (Milligan et al., 2020).

In humans, cortical layer arrangement is clearly demarcated, exemplified by clear shifts in CB1R expression between layers (Glass et al., 1997; Eggan et al., 2010; Borgan et al., 2019b; Chou et al., 2022; Durieux et al., 2022). Additionally, there are species-dependent differences in CB1R distribution, such as heightened [^3^H]CP55,940 binding in the human left hemisphere, which reflects evolutionary adaptations for refinable language circuitry (Glass et al., 1997; Bloomfield et al., 2019). Evolutionary divergences and the greater sophistication of the human brain influence differences in cytoarchitectural CB1R distribution.

### Hippocampal formation

The hippocampal formation is one of the most CB1R-dense regions across species (Herkenham et al., 1991; Glass et al., 1997; Marsicano and Lutz, 1999). CB1R protein and gene expression is greatest in interneuron-containing layers of the hippocampus and in the dentate gyrus (Herkenham et al., 1991; Glass et al., 1997; Marsicano and Lutz, 1999; Nava et al., 2001; Albayram et al., 2016; Borgan et al., 2019a; Palmisano et al., 2022). Compared to rodents, humans have increased [^3^H]CP55,940 staining and CB1R mRNA in the strata containing pyramidal and dentate cells, suggesting a species-dependent emphasis on direct modulation of these areas (Herkenham et al., 1991; Glass et al., 1997; Marsicano and Lutz, 1999; Hammond, 2015).

CB1R is present on morphologically and functionally distinct neurons, including GABAergic basket cells, GABAergic interneurons, and a small population of glutamatergic pyramidal cells (Marsicano and Lutz, 1999; Tsou et al., 1999). CB1R is expressed only on interneurons that synapse with interneurons, as indicated by the absence of parvalbumin co-expression (Marsicano and Lutz, 1999; Tsou et al., 1999). Interestingly, co-localization of calretinin (CRT), a general marker for interneurons, is greater in CB1R-immunoreactive neurons of rats than in CB1R mRNA-expressing neurons of mice (Marsicano and Lutz, 1999; Tsou et al., 1999). CRT expression in hippocampal interneurons is similar among species, suggesting differences between *CB1R* gene and protein expression underlie differences in CRT co-localization (Qi et al., 2022). In the dentate gyrus, CB1R is also expressed at glutamatergic synapses on mossy cells responsible for inhibiton of granule cells (Marsicano and Lutz, 1999; Kawamura et al., 2006; Scharfman, 2016).

Widespread cellular distribution facilitates modulation of hippocampal GABAergic neurotransmission through perisomatic synapses with principal cells and other GABAergic interneurons (Tsou et al., 1999). However, the proportion of neuronal populations expressing CB1R is unclear in humans. Elucidating subtle differences in the loci where cannabinoids act in the human hippocampus can help researchers understand the mechanisms behind unwanted memory changes seen in clinical trials. Additionally, there is a need to further understand the implications of species differences in exhibiting pain behaviors on the role of CB1R in memory processing, as prey animals, such as rodents, tend to minimize signs of pain.

### Basal ganglia

Across species, CB1R protein and gene expression in the basal ganglia is robust in the input nuclei, sparse in the intrinsic nuclei, and most abundant in the output nuclei (Herkenham et al., 1991; Glass et al., 1997; Mátyás et al., 2006). In the striatum, CB1R is expressed on GABAergic medium spiny neurons giving rise to the direct and indirect pathway (Mátyás et al., 2006). Cannabinoids are thus able to decrease activity in sensorimotor circuits, promoting movement and inhibiting competing movements (Fernandez-Ruiz and González, 2005; Lanciego et al., 2012). CB1R is also present on the axons of large parvalbumin-positive and small CRT-positive interneurons, providing another site for medium spiny neuron modulation (Tsou et al., 1998; Mátyás et al., 2006; Lanciego et al., 2012). In rats, there is a unique lateromedial gradient of striatal CB1R protein expression corresponding to regions that receive input from the primary sensory and motor cortex (Herkenham et al., 1991; Mátyás et al., 2006).

Interestingly, the rodent nucleus accumbens has sparse CB1R protein expression (Herkenham et al., 1991; Tsou et al., 1998; Mátyás et al., 2006). Although expression is low, chronic cannabinoid use produces functional tolerance at GABAergic and glutamatergic synapses (Hoffman et al., 2003). It is critical for future studies to investigate how CB1R expression and the development of tolerance differ in humans.

The globus pallidus internus in humans and the corresponding rodent entopeduncular nucleus are one of the most [^3^H]CP55,940 receptor-dense regions of the brain (Herkenham et al., 1991; Glass et al., 1997). CB1R in the output nuclei is localized to the axons of GABAergic striatonigral and pallidonigral projection fibers of the direct and indirect pathways, respectively, providing a final site for modulation before motor information is relayed to the thalamus (Herkenham et al., 1991; Glass et al., 1997; Tsou et al., 1998; Mátyás et al., 2006). [^3^H]CP55,940 binding in the entopeduncular nucleus is more intense in mice compared to rats, indicating that cannabinoids may produce a greater magnitude of locomotor changes in mice (Romero et al., 1998).

Because of the widespread expression of CB1R in the basal ganglia, both locomotion and pain behaviors should be assessed in rodent studies to ensure that a reduction in pain, not motor ability, is producing anti-nociceptive behaviors. Additionally, anatomical differences between species, such as the less distinct boundary between the rodent globus pallidus externus and entopeduncular nucleus, should be taken into consideration when conducting both preclinical research and clinical trials to increase the efficicacy of translatability (Hardman et al., 2002).

### Hypothalamus

The hypothalamus of rodents is characterized by sparse and heterogenous CB1R expression. Rats have overall greater cannabinoid radioligand binding than mice (Romero et al., 1998). In the rodent anterior complex, CB1R protein and mRNA are primarily expressed in nuclei involved in maintaining physiological homeostasis by regulating temperature, osmolarity, sleep, and arterial pressure (Herkenham et al., 1991; Tsou et al., 1998; Marsicano and Lutz, 1999; Wittmann et al., 2007; Bear et al., 2018; McKinley et al., 2021). Minimal [^3^H]CP55,940 binding, but moderate CB1R mRNA and immunoreactivity are observed in the ventral nuclei controlling satiety, metabolism, and reproduction (Herkenham et al., 1991; Tsou et al., 1998; Marsicano and Lutz, 1999; Wittmann et al., 2007; Bear et al., 2018; Song and Choi, 2023). CB1R protein expression is enriched in posterior nuclei that play a role in thermoregulation, anti-nociception, and memory (Herkenham et al., 1991; Wittmann et al., 2007; Jeong et al., 2012; Bear et al., 2018). The lateral hypothalamic area, a key regulator of orexia, sleep, stress, and motivated-behaviors, is CB1R-immunopositive, especially in the anterior portion (Tsou et al., 1998; Wittmann et al., 2007; Mickelsen et al., 2019). CB1R protein is also expressed on the external median eminence and infundibulum, creating a link with the endocrine system (Herkenham et al., 1991; Tsou et al., 1998; Wittmann et al., 2007).

In humans, CB1R protein expression has only been reported in the mamillary bodies and pituitary gland (Glass et al., 1997; Pagotto et al., 2001). Across hypothalamic nuclei, CB1R is expressed on neurons that form comparable proportions of symmetrical and asymmetrical synapses with somata and dendrites (Wittmann et al., 2007).

CB1R expression in the thalamus and hypothalamus is understudied, owing to lower overall levels of expression and the complex neuronal populations in individual nuclei. It is highly probable that humans also express CB1R in the hypothalamic nuclei, as evidenced by cannabis-induced orexia, hypothermia, and reproductive issues (Ameri, 1999; Koch et al., 2015; Lo et al., 2022). Thus, while general localization is likely conserved, it is difficult to ascertain interspecies differences critical to translatability. Additionally, variations in how thoroughly the nuclear groups are divided in studies can lead to incomparable levels of expression. Activation of CB1R in the hypothalamus by cannabinoid-based therapeutics should be avoided to prevent undesirable changes in homeostatic regulation.

### Thalamus

Across species, CB1R protein and mRNA expression is sparse in the thalamus, being localized to nuclei relaying associational information (Herkenham et al., 1991; Glass et al., 1997; Romero et al., 1998; Marsicano and Lutz, 1999; Allen Institute, 2012). The mediodorsal nucleus of rodents has the greatest CB1R protein and gene expression (Herkenham et al., 1991; Marsicano and Lutz, 1999). Increased expression in this part of the descending pain pathway may contribute to a more robust anti-nociceptive effect compared to humans. Moderate CB1R protein expression in the anterodorsal and anteroventral nuclei of rats and humans facilitates modulation of episodic memory (Glass et al., 1997; Tsou et al., 1998; Child and Benarroch, 2013).

Humans have an additional site to alter incoming somatosensory, pain, and motor information, as evidenced by low [^3^H]CP55,940 binding in the ventral nuclear group (Glass et al., 1997; Kosif, 2016). In contrast, CB1R protein and mRNA are expressed in the reticular nucleus of rodents but not humans, indicating a rodent-specific emphasis on intrathalamic modulation (Herkenham et al., 1991; Glass et al., 1997; Tsou et al., 1998; Marsicano and Lutz, 1999; Kosif, 2016). The absence of CB1R in the lateral nuclear group is conserved across species (Herkenham et al., 1991; Glass et al., 1997; Marsicano and Lutz, 1999). Among the midline nuclei, the nucleus reuniens is moderately [^3^H]CP55,940 dense in humans, whereas the paraventricular nucleus of mice has elevated CB1R mRNA levels (Glass et al., 1997; Marsicano and Lutz, 1999). [^3^H]CP55,940 binding in the intralaminar nuclei is much greater in humans than in rats (Herkenham et al., 1991; Glass et al., 1997). Heightened [^3^H]CP55,940 binding in the medial and lateral geniculate nuclei is conserved across species (Herkenham et al., 1991; Glass et al., 1997). CB1R protein and mRNA is present in the rodent habenula, predominantly in the lateral portion, indicating a species-dependent role in mediating pain and analgesia (Tsou et al., 1998; Marsicano and Lutz, 1999; Shelton et al., 2012).

Widespread CB1R expression facilitates modulation of memory encoding and retrieval, learning, awareness, higher-order functions, and affective behaviors (Fama and Sullivan, 2015; Vertes et al., 2015; Kosif, 2016). Divergences in thalamic CB1R expression may underlie key differences in the modulation of pain perception and behaviors that impact translatability (Fama and Sullivan, 2015; Kosif, 2016).

### Amygdaloid complex

The distribution of CB1R across species in the amygdaloid complex is contentious; radioligand techniques suggest a sparse to moderate, homogenous distribution while immunohistochemistry and *in situ* hybridization indicate heterogeneity (Herkenham et al., 1991; Glass et al., 1997; Romero et al., 1998; Tsou et al., 1998; Marsicano and Lutz, 1999). Enriched *CB1R* gene and protein expression were observed in the basolateral, basomedial, central, and lateral olfactory tract nuclei of rodents (Herkenham et al., 1991; Tsou et al., 1998; Marsicano and Lutz, 1999). Heightened immunostaining was also seen in the rat amygdalohippocampal area and the periamygdaloid area (Katona et al., 2001). The anterior and posterior portion of the cortical nucleus consists of CB1R-immunoreactive polymorphic neurons (Tsou et al., 1998). In rats, sparse levels of [^3^H]CP55,940-bound receptors and CB1R-immunopositive elements were present in the stria terminalis and the bed nucleus of the stria terminalis (Herkenham et al., 1991; Tsou et al., 1998). CB1R-immunoreactivity was evenly distributed within individual nuclei (Katona et al., 2001).

In rodents, CB1R-expressing amygdaloid neurons can be divided into large-diameter peptidergic GABAergic interneurons, non-peptidergic GABAergic interneurons, and glutamatergic pyramidal neurons (Marsicano and Lutz, 1999; Katona et al., 2001). Cannabinoids in the amygdala modulate anxiety- and depressive-like behaviors and the development of cue-induced fears (Mikics et al., 2006; Shen et al., 2019). Their biphasic effect on anxiety could be attributed to CB1R-positive GABAergic and glutamatergic subpopulations, which mediate anxiogenic and anxiolytic effects, respectively (Rey et al., 2012). Additionally, cannabinoids may exert an anti-emetic effect by modulating afferents from the central nucleus to the parvocellular medullary reticular formation (Herkenham et al., 1991).

Comparative levels of *CB1R* gene and protein expression in the amygdala are understudied in humans. Additionally, the affective component of pain is often overlooked when studying anti-nociception in rodents. As prey animals have evolved to hide signs of pain and distress, there are underlying differences in the affective component of pain between humans and rodents that warrant further investigation. It is critical to explore the loci at which cannabinoids modulate emotional and motivational aspects of pain to overcome obstacles in creating translatable therapeutics.

### Midbrain

Across species, midbrain CB1R expression is minimal and primarily restricted to autonomic regulatory nuclei (Herkenham et al., 1991; Glass et al., 1997; Romero et al., 1998; Tsou et al., 1998). The substantia nigra pars reticulata abundantly expresses CB1R, whereas the substantia nigra pars compacta have minimal expression (Herkenham et al., 1991; Glass et al., 1997; Tsou et al., 1998; Mátyás et al., 2006). Heightened expression in the periaqueductal gray, median raphe nucleus, and midbrain reticular formation is conserved between species (Herkenham et al., 1991; Glass et al., 1997; Tsou et al., 1998; Wilson-Poe et al., 2012). CB1R in these regions facilitates modulation of voluntary movement, the descending pain pathway, serotonergic neurotransmission, and reticular integration (Caminero and Cascella, 2024). In the rat periaqueductal gray, CB1R is predominantly localized to the soma and dendrites of presynaptic neurons (Wilson-Poe et al., 2012). Moderate immunoreactivity and [^3^H]CP55,940 binding occur in the lateral interpeduncular nucleus and superior colliculus of rats (Herkenham et al., 1991; Tsou et al., 1998). The rat oculomotor nucleus, red nucleus, ventral tegmental area, median raphe nucleus, superior colliculus, inferior colliculus, and midbrain reticular formation have sparse [^3^H]CP55,940 density and no CB1R-immunoreactivity (Herkenham et al., 1991; Tsou et al., 1998). While CB1R in humans has not been reported in several nuclei, gene and protein expression is probable owing to cannabinoid-induced changes in autonomic functions and perception (Tart, 1970; Hoch et al., 2024). Defining CB1R expression in humans across all regions of the pain pathways is crucial when reflecting on and deducing causes for lapses in translatability.

### Hindbrain

Hindbrain CB1R expression is minimal and heterogenous (Herkenham et al., 1991; Glass et al., 1997; Romero et al., 1998; Miederer et al., 2020). In humans and rats, CB1R in the hindbrain is predominantly localized to nuclei associated with autonomic functions (Herkenham et al., 1991; Glass et al., 1997; Tsou et al., 1998). Less CB1R is present in secondary sensory and motor nuclei in both species (Herkenham et al., 1991; Glass et al., 1997). Thus, cannabinoids in this region have a minor role in regulating heart rate, blood pressure, respiration, gut motility, proprioception, innocuous and noxious facial sensations, and cough, gag, and emetic reflexes across species (Hornby and Prouty, 2004; Baker and Lui, 2019; Iordanova and Reddivari, 2019). Heightened CB1R expression in the dorsal motor nucleus potentially indicates that the human gut-brain axis is more sensitive to cannabinoid modulation (Herkenham et al., 1991; Glass et al., 1997). Alternatively, the more complex diet of humans may require finer gastrointestinal regulation. Additionally, the expression of cannabinoid receptors is low in critical autonomic areas that regulate breathing, providing functional evidence that cannabinoid overdoses are less dangerous than opioid overdoses. The minimal but functionally significant population of CB1R-positive neurons in the hindbrain and their role in regulating interactions between the autonomic nervous system and the perception of pain warrant further investigation.

### Cerebellum

Cerebellar CB1R expression follows a cytoarchitectural pattern, being dense in the molecular layer, sparser in the Purkinje cell and granular layer, and minimal in the deep cerebellar nuclei (Herkenham et al., 1991; Glass et al., 1997; Romero et al., 1998; Tsou et al., 1998; Kawamura et al., 2006). CB1R in the cerebellum is predominantly expressed on the axons and terminals of GABAergic basket and stellate cells that regulate Purkinje cell output (Romero et al., 1998; Tsou et al., 1998; Ashton et al., 2004; Kawamura et al., 2006). For example, suppression of GABAergic interneurons in the molecular layer contributes to Purkinje cell depolarization-induced suppression of inhibition (Kreitzer and Regehr, 2001; Ashton et al., 2004). This mechanism facilitates cannabinoid-driven modulation of cerebellar plasticity (Lu and Mackie, 2016; Hitchcock et al., 2021). The high density of CB1R, despite the lack of proportional neuronal input, suggests a possible role of cannabinoids in the cerebellar processing of multimodal information (Herkenham et al., 1991). While overarching CB1R expression is conserved across species, increased Purkinje cell density relative to cerebellar size may influence the variation in CB1R protein expression in the molecular layer (Korbo and Andersen, 1995). It is critical to understand CB1R expression across all regions in the central nervous system implicated in higher-order processing, such as motor coordination, to minimize off-target effects and increase the success of clinical work building upon preclinical studies.

### Spinal cord

Across species and spinal cord regions, CB1R expression is greatest in the superficial dorsal horn and around the central canal (Herkenham et al., 1991; Glass et al., 1997; Tsou et al., 1998; Farquhar-Smith et al., 2000; Veress et al., 2013; Parnell et al., 2023). At the thoracic level, low levels of CB1R are present in the intermediolateral column (Glass et al., 1997; Farquhar-Smith et al., 2000). In rats, the superficial dorsal horn is characterized by two bands of heightened CB1R-immunoreactivity in lamina I and laminae IIi/III separated by sparse expression in laminae IIo (Farquhar-Smith et al., 2000; Hegyi et al., 2009). Rats have an increased superficial to deep ratio of CB1R-expression in the dorsal horn compared to humans, partially due to species-specific expression in the dorsal lateral funiculus and the superficial dorsal horn double band which has been exclusively reported in rats (Farquhar-Smith et al., 2000; Parnell et al., 2023).

CB1R is localized to axon terminals of both asymmetrical and symmetrical synapses and somatodendritic compartments (Farquhar-Smith et al., 2000; Hegyi et al., 2009; Zhang et al., 2022; Parnell et al., 2023). The *CB1R* gene is preferentially expressed on excitatory neurons in mice and humans; however, *CB1R* gene expression in mice exhibits higher neuronal subtype specificity compared to humans (Häring et al., 2018; Russ et al., 2021; Yadav et al., 2023). *CB1R* gene and protein expression is enriched in excitatory neurons associated with allodynia, mechanical nociception, noxious thermal perception, reflexive analgesia, and spinobrachial communication and inhibitory neurons involved in pain-gating, mechanical allodynia, and itch (Farquhar-Smith et al., 2000; Dableh et al., 2009; Hegyi et al., 2009; Veress et al., 2013; Häring et al., 2018; Russ et al., 2021; Zhang et al., 2022; Parnell et al., 2023; Yadav et al., 2023).

CB1R in the spinal cord is expressed on central terminals, excitatory interneurons, inhibitory interneurons, and spinobrachial projections, allowing for polymodal modulation of both first and second-order neurons in ascending and descending pathways. Understanding how nociceptive perception and transmission are impacted by species divergences in CB1R expression is critical for translatability. Differences in neuronal subtypes may underlie the distinctive organization of CB1R seen in rats, further contributing to challenges in translatability. Additionally, neuronal subtypes found in rodents and humans do not correlate one-to-one. Further development and utilization of harmonized spinal cord atlases can elucidate differences in the distribution and proportion of neurons modulated by the endocannabinoid system.

## CB1R Expression throughout Regions of the Peripheral Nervous System across Different Species

**Dorsal root ganglia**

**Figure 4:**
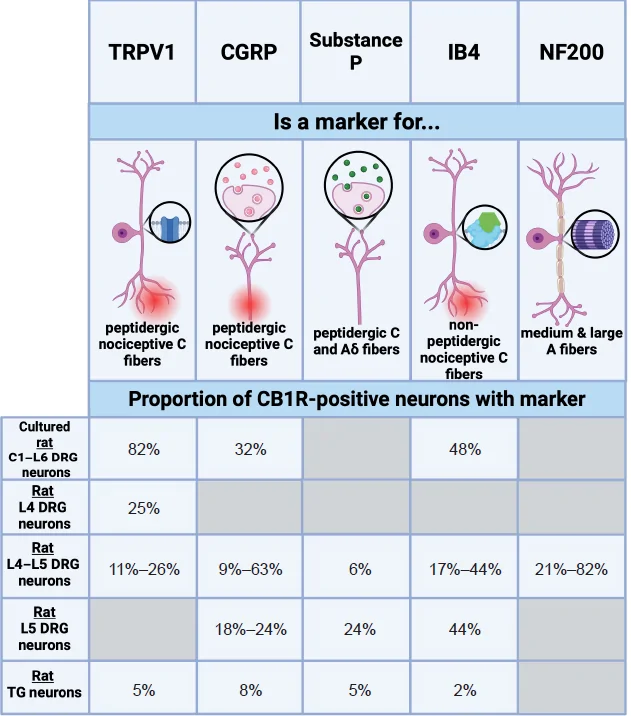
Graphical representation of the co-localization of CB1R with common neuronal markers in the peripheral nervous system. A grey box indicates that the available literature does not report the proportion of CB1R-positive neurons expressing the specified marker. Created with BioRender.com. CB1R: Cannabinoid receptor 1; CGRP: calcitonin gene-related peptide; IB4: griffonia simplicifolia isolectin B4; NF200: neurofilament 200; TRPV1: transient receptor potential vallinoid 1.

Expression of CB1R in the dorsal root ganglia (DRG) differs between spinal levels and species (Horvath et al., 2011; Klar et al., 2015; Li and Coffield, 2016; Luo et al., 2020; Chen et al., 2025). The proportion of CB1R on all neurons and specific neuronal subpopulations remains debated (**Figure 4**; Spiegelman, 2010; Fichna et al., 2013; Morena et al., 2015; Davis et al., 2018; Ho et al., 2023). A similar percentage of CB1R-expressing DRG neurons has been reported in rodents and humans; however, human *CB1R* gene expression is significantly less abundant (Ahluwalia et al., 2002; Bridges et al., 2003; Mitrirattanakul et al., 2006; Veress et al., 2013; Ray et al., 2018; Ford et al., 2021). CB1Rs are transported from DRG somata to peripheral terminals where they modulate afferent signaling, offering a potential pain relief strategy without engaging the central nervous system (Hohmann and Herkenham, 1999).

The degree of co-expression of CB1R and various neuronal markers in the DRG is also inconsistent between species and techniques. Across species, CB1R is expressed on a broad range of small and medium diameter neurons, including peptidergic and non-peptidergic nociceptors, mechanoreceptors, and proprioceptors (Ahluwalia et al., 2002; Bridges et al., 2003; Mitrirattanakul et al., 2006; Tavares-Ferreira et al., 2022; Yu et al., 2024). Humans additionally express CB1R mRNA in a population of Aδ high-threshold mechanoreceptors and somatostatin-positive thermal nociceptive C-fibers (Tavares-Ferreira et al., 2022). CB1R expression in rodents is highly associated with peptidergic C-fibers (Bhuiyan et al., 2024). Human sensory neurons show a broader overlap of markers, making it difficult to classify them into the distinct peptidergic and non-peptidergic categories common in rodent models (Tavares-Ferreira et al., 2022; Yu et al., 2024). Additionally, regional variation within species adds another layer of complexity; in mice, CB1R mRNA is notably expressed in lumbar somatostatin–positive nociceptors but not in those of the cervical region (Yu et al., 2024). Variability in neuronal subtypes, marker expression, and receptor localization across species limits translational confidence.

Despite these differences, efforts have been made to investigate the presence of CB1R in peripheral sensory neurons as a therapeutic target (Rangari et al., 2025). Identifying which sensory populations cannabinoids target, and how those populations differ across species, is crucial for closing the translational gap. Only by achieving this understanding can we fully assess therapeutic potential of CB1R and address long-standing public misconceptions that stem not from scientific failure, but from the biological complexity we are just beginning to uncover.

### Trigeminal ganglion

CB1R expression in the trigeminal ganglion has been reported at both the mRNA and protein levels, though binding studies indicate sparse receptor presence in rat ganglia and nerves (Herkenham et al., 1991). CB1R mRNA is predominantly found in medium-to-large diameter neurons, especially those projecting to the mandibular and maxillary branches (Price et al., 2003). These neurons largely co-express NF200, marking them as amyloid-beta fibers, while showing minimal overlap with nociceptive markers. In humans and rodents, CB1R mRNA is conserved in Aδ low-threshold mechanoreceptors; however, rodents also exhibit expression in Aδ high-threshold mechanoreceptors and injury-responsive populations, suggesting a broader functional role and potentially more robust anti-nociceptive effects. These interspecies differences in receptor distribution help explain why preclinical studies often show promising outcomes that are not always replicated in clinical trials. Recognizing these differences enables us to refine cannabinoid-based therapies, clarify misinterpretations of studies, and better communicate their true potential for providing pain relief through peripheral mechanisms.

## CB2R Expression throughout Various Tissue and Cell Types

While CB1R has been found on cells and in tissue outside of the nervous system, CB2R is considered a potential target for pain therapeutics owing to its ability to produce anti-nociception through neuroimmune interactions without central nervous system activation (Galiègue et al., 1995; Ryberg et al., 2005; Amaya et al., 2006; Atwood and Mackie, 2010; Wilson-Poe et al., 2012; Veress et al., 2013). Historical studies report CB2R mRNA in peripheral immune cells and lymphoid tissue, but not the nervous system (Galiègue et al., 1995). Recently, CB2R mRNA has been observed in rodent cortical microglia, GABAergic striatal interneurons, hippocampal interneurons and pyramidal cells, hypothalamus, nucleus accumbens, amygdala, cerebellum, brainstem, retina, and CD11b-positive microglia of the spinal cord (Grabon et al., 2023). CB2R-immunoreactivity has been detected in neurons of the ventral tegmental area, red nucleus, dorsal motor nucleus of the vagus, nucleus ambiguous, spinal trigeminal nucleus, CA1, dentate gyrus, and retina and in astrocytes in the ventral tegmental area of rodents (Grabon et al., 2023).

CB2R antibodies have been validated by western blotting and blocking by an immunizing peptide, but not in a full CB2R knockout animal due to no such strain existing (Grabon et al., 2023). Because the probes created to detect CB2R mRNA via *in situ* hybridization should be highly specific, it is unlikely that the CB2R mRNA signal is from non-specific binding of other gene sequences. It is possible that brain regions with reported CB2R mRNA and protein expression do express CB2R to some degree, but further validation studies are required to ensure consistent and precise detection.

## Changes in Expression in Response to Pathological Conditions

Pathology-induced changes in expression of cannabinoid receptors may underlie mechanisms in which the endocannabinoid system adapts to facilitate more efficacious modulation. Conflicting results in preclinical and clinical trials may be due to conflating noncongruent types of pain and species divergences in basal expression. Understanding how basal and altered expression of cannabinoid receptors fits into the mechanistic framework of multiple types of pain is crucial for overcoming obstacles in translating cannabinoid-based therapeutics.

The time course of changes in CB1R expression in rodents and man could provide insight into the specific window in which the endocannabinoid system can yield the greatest anti-nociceptive benefit. Peripheral neuropathic pain increased CB1R expression in the dorsal horn of rats, peaking at 14 days post injury (Lim et al., 2003). CB1R mRNA and protein are also upregulated in DRGs proximal to the level of injury (Mitrirattanakul et al., 2006). Upregulation of CB1R adjacent to peripheral nerve injury serves a protective function to regulate pain and inflammation. Further consideration into the contributions of endocannabinoid signaling and the level of damage should be taken to assess how changes in CB1R expression can be harnessed for therapeutic purposes. Additionally, increased TRPV1 colocalization is observed in L4 DRGs whereas IB4 and calcitonin gene-related peptide co-localization is decreased in L5 DRGs (Mitrirattanakul et al., 2006). Thus, increased expression is due to increases in the number of receptors in neurons, rather than a result of a phenotype shift. CB1R epigenetic regulation can silence gene expression after weeks after neuropathic injury as a result of histone modification (Luo et al., 2020). The incidence of changes in epigenetic expression and receptor functionality due to post-translational modifications may differ between species, offering another explanation as to why the anti-nociceptive effect of cannabinoids is not consistently seen in clinical trials.

**Figure 5:**
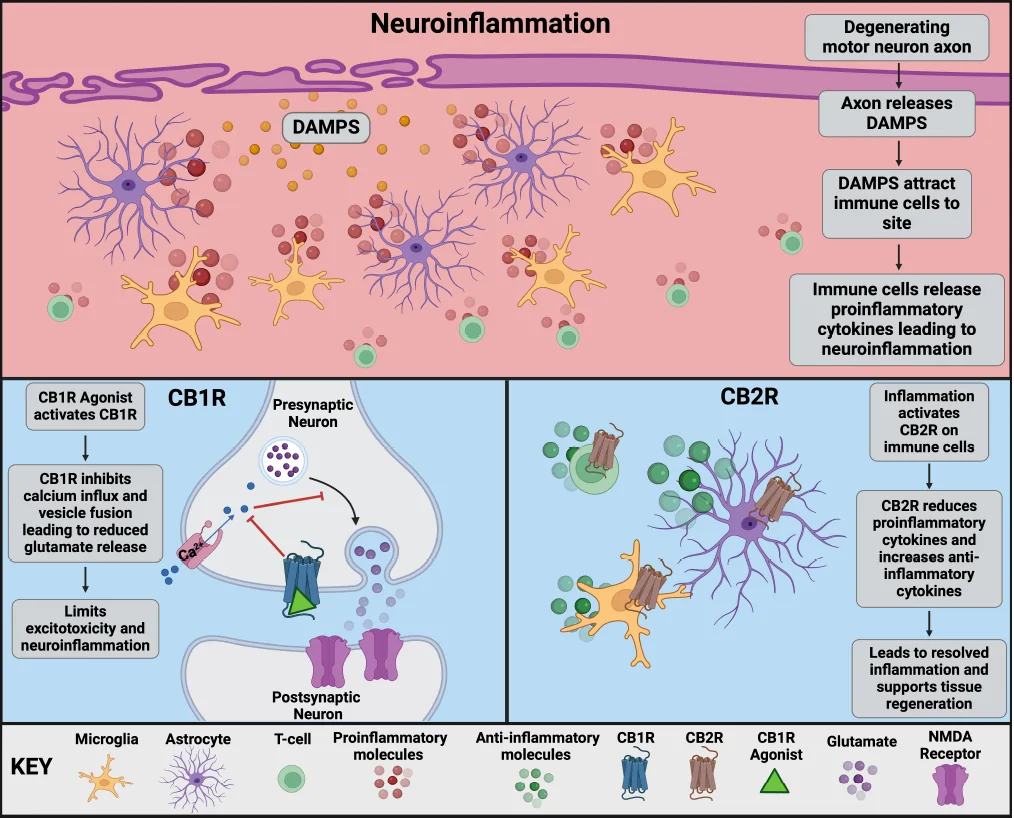
Graphical representation of the complementary roles of CB1R and CB2R in regulating neuroinflammation in the central nervous system. The top section, indicated in red, shows the process of neuroinflammation, where a degenerating neuron releases DAMPS to attract immune cells to the site of injury. These accumulating immune cells release proinflammatory cytokines, which lead to neuroinflammation. The bottom left section shows CB1R expressed on presynaptic terminals. After activation by an agonist, CB1R inhibits voltage-gated calcium channels, which reduces calcium influx into the presynaptic terminal. This leads to a decrease in glutamate vesicle release into the synaptic cleft, preventing the overstimulation of postsynaptic terminals. This limits excitotoxicity and directly reduces neuroinflammatory responses. The bottom right section shows CB2Rs role in neuroinflammation. CB2R expressed on immune cells, specifically microglia, T-cells, and astrocytes regulates the immune response during neuronal injury. CB2R activation changes the immune cells from proinflammatory to an anti-inflammatory state. This transition inhibits the release of proinflammatory cytokines and promotes the release of anti-inflammatory mediators, leading to the dissipation of the inflammation. Created with BioRender.com. CB1R: Cannabinoid receptor 1; CB2R: cannabinoid receptor 2; DAMPS: damage-associated molecular patterns; NMDA: N-methyl-D-aspartate.

Alterations in cannabinoid receptor expression also occur during acute and chronic inflammatory pain (**Figure 5**; Komine and Yamanaka, 2015; Szczesniak et al., 2017). Cannabinoid receptors and cytokines have a bidirectional relationship, whereby cannabinoid receptors on immune cells modulate cytokine levels and cytokines modulate cannabinoid receptor expression (Jean‐Gilles et al., 2015). Pro-inflammatory cytokines induce CB1R and CB2R mRNA expression upregulation through the NF-κB pathway in human whole blood, peripheral blood mononuclear cells, and T-cells (Jean‐Gilles et al., 2015). Additionally, increased concentration of pro-inflammatory cytokines in patients with multiple sclerosis is correlated with increases in CB1R and CB2R mRNA expression in blood (Jean‐Gilles et al., 2015). Intraplantar injection of complete Freund’s adjuvant increased CB1R mRNA and protein in rat L4–L5 DRGs 1 day post injection before returning to basal levels 7 days post injection (Amaya et al., 2006). This upregulation of CB1R is specific to small diameter, TRPV1-immunopositive neurons in the DRG and nerve fibers of the dermis, suggesting inflammation results in increases in CB1R expression in both primary afferent neurons and peripheral axons (Amaya et al., 2006). CB1R expression in immune cells warrants further investigation, as cannabinoid-regulated modulation of neuroimmune interactions could influence anti-nociceptive mechanisms and the transition from acute to chronic pain and expand broader therapeutic potential of CB1R.

Pain often has other comorbid pathologies, resulting changes in cannabinoid expression must also be considered to accurately attribute changes in expression to pain. For example, acute stress has been reported to reduce CB1R mRNA and increase phosphorylation, leading to receptor inactivation and internalization, thus reducing the effectiveness of cannabinoids (Xing et al., 2011). Studying the effect of stress on CB1R expression and resulting cannabinoid effectiveness in humans may provide insight into overcoming this obstacle in cannabinoid research.

## Discussion

The distribution of *CB1R* gene and protein expression is generally conserved across species. The greatest density of CB1R is found in the globus pallidus internus, substantia nigra pars reticulata, molecular layer of the cerebellum (Herkenham et al., 1990; Glass et al., 1997; Romero et al., 1998; Marsicano and Lutz, 1999). CB1R is highly concentrated in the hippocampus, dentate gyrus, and striatum (Herkenham et al., 1990; Glass et al., 1997; Romero et al., 1998; Marsicano and Lutz, 1999). Regions of moderate expression include the neocortex, basal amygdala, hypothalamus, periaqueductal gray, nucleus of the solitary tract, and laminae I–III and X of the spinal cord (Herkenham et al., 1990; Glass et al., 1997; Romero et al., 1998; Marsicano and Lutz, 1999). The thalamus and brainstem have the lowest density of CB1R (Herkenham et al., 1990; Glass et al., 1997; Romero et al., 1998; Marsicano and Lutz, 1999). Thus, CB1R is present in nervous tissue associated with motor function, information processing, emotional processing, memory formation, encoding of sensory information, autonomic processes, and pain modulation. This widespread distribution allows cannabinoids to produce CB1R-mediated biological and psychoactive effects across species.

Species divergences arise in the level of CB1R expression and functional cellular localization. This is especially salient in the spinal cord and DRG due to the non-homologous nature of sensory neuron populations, especially those implicated in nociception. In rodents, peptidergic nociceptors have clearly defined gene expression profiles, facilitating the use of peptidergic markers such as calcitonin gene-related peptide and substance P to distinguish them from nonpeptidergic nociceptors (Rostock et al., 2018; Tavares-Ferreira et al., 2022; Yu et al., 2024). However, human nociceptors broadly express both peptidergic and nonpeptidergic markers, suggesting a clear division does not exist in humans (Rostock et al., 2018; Tavares-Ferreira et al., 2022; Yu et al., 2024). Additionally, greater diversity of gene expression is seen in mouse C-fiber nociceptor subtypes, whereas humans have more molecularly distinct A fiber nociceptor subtypes (Yu et al., 2024). Understanding the shifts in nociceptor populations between rodents and humans and how it affects functional CB1R localization is critical for overcoming the obstacle in translating the positive preclinical findings of the anti-nociceptive action of cannabinoids.

There are often discrepancies in cannabinoid receptor protein and gene expression between studies using the same species and similar techniques, making it difficult to ascertain the precise distribution and expression proportion of CB1R and CB2R. Commercially available CB1R and CB2R antibodies lack specificity, leading to inaccurate measures of expression (Grimsey et al., 2008; Zhang et al., 2019). These antibodies are also unable to detect isoforms of the receptor that are present at low levels, such as heterodimers formed with other G protein-coupled receptors, post-translational modified species, and splice variants (Mackie, 2005; Ryberg et al., 2005; Grimsey et al., 2008; Lu and Mackie, 2021). Future studies should rigorously validate their antibodies with the use of proper controls. Optimization of tissue preparation, blocking conditions, incubation conditions, and reagents should also be performed and reported for replicability. Additionally, because CB1R is primarily synthesized in the soma and transported to peripheral terminals, ISH and qPCR techniques cannot estimate axonal and synaptic expression levels (Hohmann and Herkenham, 1999). However, the greater sensitivity of these techniques allows for the detection of low levels of mRNA, which led to the discovery of CB1R and CB2R outside of where they are conventionally expressed (Galiègue et al., 1995). Precisely understanding expression patterns across tissues and species will aid in minimizing disparities between preclinical and clinical translatability. Lastly, the limited spatial resolution of confocal microscopy may also impact the quantification of CB1R expression and co-localization (Hegyi et al., 2009).

The anatomical and cellular loci of CB1R necessary for producing anti-nociception are currently unclear, creating another barrier in translatability. Transgenic animal models can be utilized to determine the function of CB1R by selectively ablating or restricting expression in a specific cell type (Howlett et al., 2002). For example, CB1R on superficial dorsal horn neurons was found to have a role in mediating neuropathic mechanical allodynia by using adeno-associated virus vectors containing shRNA to selectively knock-down CB1R in rats (Sueto et al., 2025). Cannabinoids acting on CB1R small diameter fibers in the DRG have also been implicated in reducing mechanical allodynia and thermal hyperalgesia, as evidenced in mouse models with conditional expression of CB1R on Na_v_1.8 sensory neurons (Agarwal et al., 2007). However, while transgenic mouse models aid in uncovering pathway functions, considerations of sequences of promoter regions and targeted alleles are crucial when translating work from preclinical rodent models to humans. Furthermore, optogenetics is a powerful tool for examining the role of cannabinoids in neuronal circuitry by spatially and temporally controlling CB1R expression.

The next step in improving translatability of cannabinoid therapeutics is definitively determining the cellular population that produces CB1R-mediated anti-nociception. Once the loci have been identified, species differences in that cellular population, whether proportion, overall gene expression, or *CB1R* gene and protein expression, must be elucidated using comparative transcriptomic atlases. Only then can targeted cannabinoid therapeutics using the harmonized knowledge of rodent and human nociceptive perception and sensation overcome current barriers in translatability.

To overcome the challenge of low CB1R expression in peripheral targets, a promising approach involves using a CB1R agonist that targets a cryptic binding pocket recently identified in the receptor (Rangari et al., 2025). This enables peripheral and functionally selective activation, enhancing potency while minimizing central effects. Leveraging this structural insight offers a rational design path toward safer, more effective cannabinoid therapies.

Clinically, the distinct anatomical distribution of CB1R and CB2R informs both the potential and limitations of cannabinoid therapeutics. For example, the high CB1R density in brain regions associated with psychoactivity complicates systemic administration due to adverse effects, while lower receptor levels in peripheral tissues present challenges for achieving effective analgesia without central side effects. Understanding these patterns guides the development of drugs that preferentially target peripheral receptors or specific receptor subtypes, minimizing adverse effects.

While there is much work to do in comprehensively understanding CB1R expression across the cellular, tissue, and species levels, meta-analysis of open-source sequencing data is an efficient starting point. The discussed anatomical distribution, cellular localization, and functionality of cannabinoid receptors throughout the body lays down foundational knowledge in interpreting and designing research through the lens of translatability. There is an increasing body of evidence that cannabinoids are effective in relieving chronic pain in preclinical models. To address the current concerns of low-quality clinical evidence, minimal translatability, and lack of knowledge on CB1R functionality, the guidelines highlighted in this review must be considered in future cannabinoid research. Rigorous, transparent, and optimized research utilizing contemporary techniques and knowledge will pave the way towards the increased translatability required to develop a cannabinoid therapeutic that alleviates pain and the quality-of-life reduction caused by chronic pain.

## Data Availability

*Not applicable.*
